# Phenotypic Traits, Hormonal Distribution, and Metabolite Profiling of *Isatis indigotica* Seeds from 21 Samples in China: A Traditional Chinese Medicinal Herb

**DOI:** 10.3390/plants14071096

**Published:** 2025-04-01

**Authors:** Lunyan Wang, Jia Liu, Yilun Dong, Yanan Gao, Xiangyu Xing, Tianyue Cong, Li Meng, Wanru Li, Xinyu Li, Viktar Lemiasheuski, Chunying Zheng, Yang Liu

**Affiliations:** 1 Engineering Research Center of Agricultural Microbiology Technology, Ministry of Education & Heilongjiang Provincial Key Laboratory of Ecological Restoration and Resource Utilization for Cold Region & Heilongjiang Provincial Key Laboratory of Plant Genetic Engineering and Biological Fermentation Engineering for Cold Region & School of Life Sciences, Heilongjiang University, Harbin 150080, China; wunglunyan@163.com (L.W.); lixinyu2434@163.com (X.L.); 2 Key Laboratory of Soybean Molecular Design Breeding, State Key Laboratory of Black Soils Conservation and Utilization, Northeast Institute of Geography and Agroecology, Chinese Academy of Sciences, Harbin 150081, China; liujia@iga.ac.cn; 3 School of Media, Harbin Normal University, Harbin 150080, China; 4 Department of Biochemistry and Bioinformatics, Polessky State University, Pushkina Str. 4, 225710 Pinsk, Belarus

**Keywords:** Chinese medicinal plant, geographical origin, metabolic differences, endogenous hormones, seed morphology

## Abstract

“Banlangen” (*Isatis indigotica* Fort., indigowoad root) is the dried root of a plant in the Cruciferae (Brassicaceae) family, which has been cultivated in China for over 2000 years. This herb has significant medicinal value and played an important therapeutic role during the COVID-19 pandemic. *Isatis indigotica* is widely cultivated in China, with varying seed quality across different regions. Investigating the seed quality and distribution of endogenous metabolites of *Isatis indigotica* from various regions is of great significance for its efficient utilization. In this study, 21 samples from *Isatis indigotica* seeds were collected from 15 different regions of China and analyzed using gas chromatography-mass spectrometry (GC-MS) and liquid chromatography-mass spectrometry (LC-MS). A systematic analysis was conducted on seed phenotypes, germination rate, endogenous metabolites, and hormones. The results showed that the germplasm traits of *Isatis indigotica* from northern China were generally superior to those from southern China, with seed size positively correlated with the accumulation of sugars (D-allose, D-(+)-cellobiose, maltitol, α-D-glucopyranoside, d-galactose, maltose, D-(−)-fructose, and galactitol) and organic acids (hexadecanoic acid, benzoic acid, propanedioic acid, butanedioic acid, and cinnamic acid). Additionally, seed germination in the samples from SuQian and Shanxi was closely related to hormone distribution (gibberellic acid and abscisic acid). This study provides valuable data to support the evaluation of seeds from different sources of medicinal plants and has important implications for the cultivation and identification of *Isatis indigotica* varieties.

## 1. Introduction

*Isatis indigotica* has a long history of cultivation in China and is grown in a wide range of areas. In the 1980s, provinces such as Hebei, Jiangsu, Anhui, and Henan were the main growing regions [1]. However, over the past two decades, the planting areas have changed significantly, with Shanxi gradually becoming the primary producer of *I. indigotica* seeds, while emerging planting areas have developed in Gansu and Heilongjiang. In 2019, *I. indigotica* was planted over approximately 11,000 hectares in China. The COVID-19 pandemic caused this to increase to 17,000 hectares in 2020. Despite the increased planting area, the germplasm resources of *I. indigotica* in China remain mixed, and the breeding of varieties lacks systematization and scale. Since *I. indigotica* primarily propagates through seeds, the selection of high-quality seeds is crucial for improving cultivation quality [2].

In agricultural production, the quality of seed germination is vital for the uniform growth and development of crops [3]. Seeds with high viability and sufficient nutrients provide the necessary space for plant growth, while poorly germinated seeds may lead to uneven seedling growth, resulting in potential economic losses [4]. In the study of herbal medicine quality, seed size and germination characteristics are crucial factors that determine the production quality and stability of medicinal materials. Seed size directly influences the seeds’ physiological and biochemical properties, including the storage of nutrients in the endosperm, seed vigor, and the initial growth advantage of seedlings. Germination capacity, on the other hand, determines the emergence rate of plants, growth rate, and the eventual accumulation of medicinal components [5].

Seed length and width are two additional key indicators for assessing seed quality, as seed size is an important factor influencing seedling vigor and seed yield [6]. Generally, larger seeds tend to store more nutrients, such as starch, proteins, and lipids, which provide energy support for early seedling growth, thereby conferring stronger stress resistance and competitiveness. In crops, rice (*Oryza sativa* L.) seed germination and seeding vigor are positively correlated with seed size [7]. In the case of the Chinese herb Ginseng (*Panax ginseng Meyer*), larger seeds have been found to exhibit higher germination rates, faster seedling growth, and more robust rhizome development [8]. This directly impacts the yield and quality of the medicinal part, which is the rhizome. Therefore, in the cultivation of medicinal herbs, screening for appropriate seed sizes is of significant importance for enhancing the yield and efficacy of medicinal materials. Seed size also affects seed storage longevity and germination time. Larger seeds, containing more stored substances, can maintain their viability for longer periods under certain conditions. In contrast, smaller seeds are more susceptible to environmental factors such as water loss, oxidation, and microbial infection, which can lead to a decline in viability. For example, in Panax ginseng (commonly known as ginseng), larger seeds exhibit higher germination rates during long-term storage, possibly due to their stronger antioxidant capacity and lower metabolic rates.

Seed germination is a crucial stage in the plant life cycle, regulated by a variety of internal and external factors. External factors include temperature, water availability, light, and oxygen, while internal factors mainly involve sugars, organic acids, hormones, enzyme activities, and gene regulatory mechanisms within the seed. Sugars serve as the primary energy source for embryo development and seedling growth. During seed germination, sugars not only provide energy but also participate in signal transduction and the regulation of seed dormancy release. For instance, soluble sugars such as sucrose, glucose, and fructose influence the germination rate, while starch content determines the energy supply for embryo development. In addition, organic acids play important roles in respiration metabolism and antioxidant processes [9]. For example, citric acid and malic acid participate in the tricarboxylic acid (TCA) cycle to provide energy for germination. Moreover, certain organic acids possess stress-resistance functions, which enable seeds to survive under adverse environmental conditions. The levels of these compounds are critical for seed germination [10,11].

Additionally, endogenous hormones in plants play crucial regulatory roles in seed growth, development, and stress resistance. Hormones such as auxins, cytokinins, gibberellins, and abscisic acid (ABA) in seeds exert distinct functions during germination and development [12]. Auxins play a key role in promoting seed germination and root growth [12]; cytokinins primarily facilitate cell division and expansion in seeds [13,14]; gibberellins are involved in stimulating seed germination and overcoming dormancy [15,16]. In contrast, ABA mainly regulates seed dormancy and stress resistance, exerting important protective functions under adverse conditions such as drought and low temperature [14,16,17]. For example, seeds from certain sources may exhibit higher germination rates and growth potential due to elevated levels of auxins or gibberellins. In contrast, seeds from other sources may possess stronger stress resistance under unfavorable conditions due to higher ABA content [16].

This study collected 21 germplasm resources of *I. indigotica* (Banlangen) from 15 regions across China to explore the advantages of different local germplasms and investigate the effects of seed metabolite content and hormone accumulation on seed phenotype and germplasm germination. Using GC-MS and LC-MS techniques, the study systematically compared the metabolite content and hormone levels of seeds from different regions and analyzed their impact on the phenotype and germination of *I. indigotica* seeds, thereby further revealing the characteristics of *I. indigotica* germplasm resources. This research provides valuable information for the study and utilization of cultivated germplasm while also offering a reference for the development and utilization of the natural resources of this species. Furthermore, investigating the endogenous metabolites and physiological characteristics of *I. indigotica* seeds from different regions is of great significance for optimizing seed quality, improving planting efficiency, achieving high-quality breeding, and enhancing the uniformity and adaptability of medicinal plant growth.

## 2. Results

### 2.1. Phenotype of I. indigotica Seeds Under Different Regional Conditions

**Figure 1:**
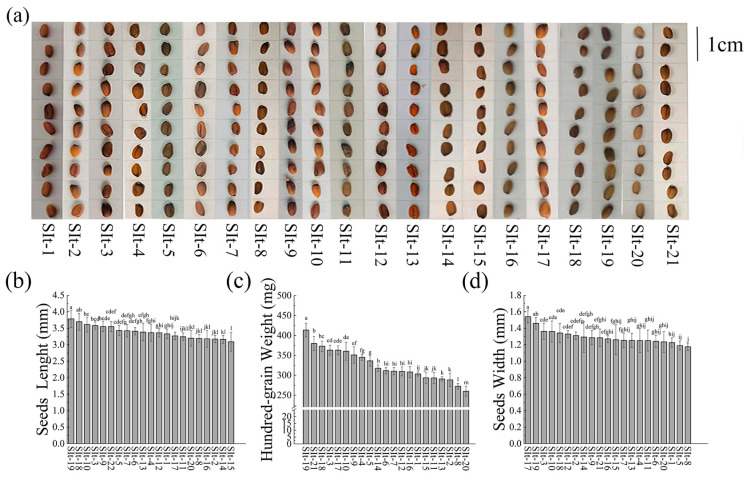
*I. indigotica* root seed phenotypes and physical data: (**a**) Seed appearance; (**b**) Seed length; (**c**) Hundred-grain weight; (**d**) Seed width. Different lowercase letters on the bar indicate significant differences among the means in different treatments (*p* < 0.05). Here, the X-axis includes the number of the collected sample of *I. indigotica*, and the specific number corresponding to the sample can be seen in Table 1.

In this study, to analyze the morphological characteristics of seeds from different sources, ten seeds were randomly selected from each region and placed on a grid board under standardized conditions for imaging and recording (Figure 1a). Comparisons revealed significant differences in seed size, plumpness, and morphological uniformity among different samples. Visually, seeds from SIt-1, SIt-3, SIt-20, and SIt-18 exhibited higher plumpness and more uniform morphology, suggesting better developmental status. In contrast, seeds were from 3.09 ± 0.29 mm to 3.78 ± 0.26 mm in length, with an average length of 3.38 ± 0.19 mm. Seeds from Xingning, Guangdong Province, had the shortest length (3.09 ± 0.29 mm), whereas those from Datong District, Daqing City, Heilongjiang Province, had the longest length (3.78 ± 0.26 mm), representing a 22.3% increase compared to the former (Figure 1b). Regarding a further analysis of seed weight, seeds from SIt-2, SIt-14, SIt-16, and SIt-21 were relatively smaller and exhibited a certain degree of morphological variability. Notably, the 100-seed weight of SIt-20 was significantly higher than that of SIt-21 and SIt-16 by 59% and 41.1%, respectively (Figure 1c), indicating that SIt-20 seeds had a greater individual weight, which may correspond to a higher storage compound content, potentially influencing germination capacity and early growth performance. These length variations may be associated with growth environment factors such as climate, soil nutrients, growth period, and genetics. In terms of seed width, measurements ranged from 1.17 ± 0.05 mm to 1.54 ± 0.08 mm, with an average width of 1.30 ± 0.08 mm. Seeds from Shanxi Province had the largest average width (1.54 ± 0.088 mm), which was 1.33 times greater than those with the smallest width (Figure 1d).

### 2.2. Metabolite Detection in I. indigotica Seeds from Different Regions

**Figure 2:**
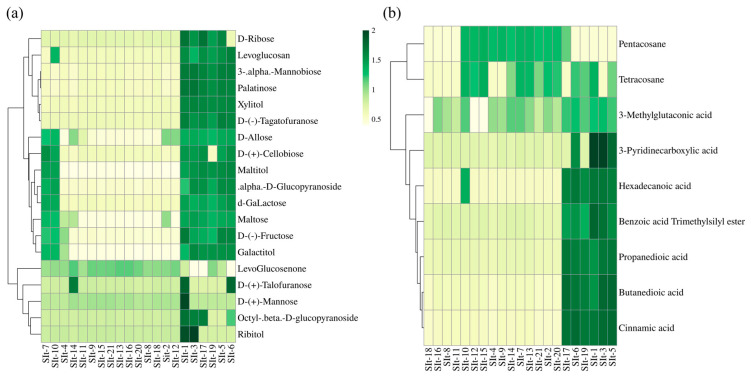
Clustering heat map analysis of *I. indigotica* seeds from different regions: (**a**) 19 saccharides and other compounds of *I. indigotica* seeds; (**b**) nine organic acids and other compounds of indigowoad root seeds. Here, the X-axis includes the number of the collected sample of *I. indigotica*, and the specific number corresponding to the sample can be seen in Table 1.

Carbohydrate metabolites and organic acids compounds, as endogenous metabolites of seeds, participate in the seed development process [12,18]. In this study, gas chromatography-mass spectrometry (GC-MS) was used to analyze the carbohydrate and organic acids compounds in *L. indigotica* seeds from 21 different sources. In this study, 19 sugar compounds were identified in *L. indigotica* seeds, and a cluster analysis was performed to explore their distribution patterns and potential biological significance. The results indicated that these sugar compounds could be classified into three major groups, with significant differences in accumulation levels among different seed samples (Figure 2a). This suggests that the synthesis and accumulation of sugars are influenced by seed origin. Our findings revealed that sugar compound accumulation was particularly high in seed samples SIt-1, SIt-3, SIt-5, SIt-6, SIt-17, and SIt-19. These samples were especially enriched in D-allose, D-(+)-cellobiose, maltitol, α-D-glucopyranoside, d-galactose, maltose, D-(−)-fructose, and galactitol. These sugars may play crucial roles in seed development, such as serving as energy reserves, regulating osmotic pressure, and participating in antioxidant and stress response mechanisms [19,20]. In addition, notable accumulation of these sugars was also detected in samples SIt-7 and SIt-10, suggesting the presence of similar regulatory mechanisms in sugar metabolism. Further analysis demonstrated that D-ribose, 3-α-mannobiose, xylitol, D-(−)-tagatofuranose, D-(+)-mannose, D-(+)-talofuranose, ribitol, octyl-β-D-glucopyranoside, and D-allose were significantly accumulated in SIt-1 and SIt-3. These compounds may be closely associated with cell wall synthesis, signal transduction, and antioxidative capacity, indicating that these seeds might exhibit higher physiological activity or enhanced environmental adaptability. In contrast, seed samples SIt-4, SIt-14, SIt-11, SIt-9, SIt-15, SIt-21, SIt-13, SIt-16, SIt-20, SIt-8, SIt-18, SIt-2, and SIt-12 exhibited relatively lower sugar accumulation, which could be attributed to weaker sugar metabolism activity or differences in energy allocation across metabolic pathways. In this study, a total of 9 organic acids were identified, and their distribution patterns in different seed samples were analyzed using cluster analysis (Figure 2b). The results showed significant differences in the accumulation levels of organic acids among different seed samples. Notably, the seed samples SIt-1, SIt-3, SIt-5, SIt-6, SIt-17, and SIt-19 exhibited higher levels of organic acids, primarily including hexadecanoic acid, benzoic acid, propanedioic acid, butanedioic acid, and cinnamic acid. In addition, 3-pyridinecarboxylic acid was significantly accumulated in the SIt-1 sample. Furthermore, three specific compounds, 3-methylglutaconic acid, tetracosane, and pentacosane, were predominantly accumulated in the germplasms SIt-10, SIt-12, SIt-15, SIt-4, SIt-9, SIt-10, SIt-14, SIt-7, SIt-13, SIt-21, SIt-2, and SIt-20. These results indicate significant differences in sugar metabolism and organic acid accumulation among seeds from different origins. Notably, seed samples SIt-1, SIt-3, SIt-5, SIt-6, SIt-17, and SIt-19 exhibited higher levels of metabolites, surpassing those from other regions. These germplasms may possess superior advantages in seed energy supply, antioxidant capacity, and environmental adaptability.

### 2.3. Analysis of Hormone Content in the I. indigotica Seed from Different Regions

**Figure 3:**
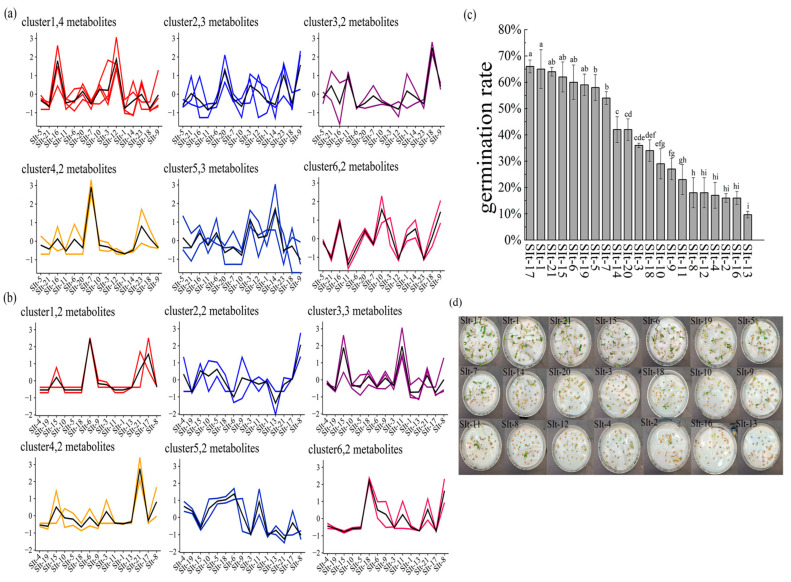
K-mean clusters of the expression profiles of hormones in 15 different regions of *I. indigotica* root seeds and germination rates: (**a**) 13 differential gibberellin, abscisic acid, and zeatin; (**b**) 16 differential cytokinins and cell growth factors hormones; (**c**) Germination rates; (**d**) Photographs of germination. Different lowercase letters on the bar indicate significant differences among the means in different treatments (*p* < 0.05). Here the X-axis includes the number of the collected sample of *I. indigotica*, and the specific number corresponding to the sample can be seen in Table 1.

Based on seed phenotypic traits and germination capacity, the hormones were categorized into two major groups: one group regulates seed size, and the other influences seed germination ability. Through analysis, hormones involved in regulating seed size were identified, including lignans, dihydrozeatin riboside, cis-zeatin riboside, trans-zeatin riboside, 6-benzylaminopurine, and scopolamine. Using K-means clustering analysis, the first group of hormones was divided into six clusters (Figure 3a). Lignans, dihydrozeatin riboside, cis-zeatin riboside, and trans-zeatin riboside belong to the first cluster. These hormones exhibit higher levels in SIt-11 and SIt-15, while lower levels are observed in SIt-18 and SIt-7. Additionally, 6-benzylaminopurine and scopolamine belong to the third cluster, with higher levels in SIt-11 and SIt-15, and lower levels in SIt-18 and SIt-6. Furthermore, K-means clustering analysis of hormones associated with seed germination (Figure 3b) revealed that Cluster 1 contains two hormones—gibberellin A1 and cis-zeatin—which exhibited higher levels in the SIt-6, SIt-21, and SIt-17 samples. In Cluster 5, gibberellins A4 and A7 were found at higher levels in the BIt-6 and BIt-17 seeds, suggesting that these hormones play a promotive role in the germination process [21] of these two seed varieties. In Cluster 6, gibberellin A9 levels were higher in the BIt-6 and BIt-21 seeds. As a lesser-studied member of the gibberellin family, gibberellin A9 may play a supplementary role in the germination process of these specific seeds, particularly in regulating the growth of the embryo during the later stages of germination [15]. Notably, in Cluster 2, all three samples exhibited lower levels of gibberellin A3 and abscisic acid (ABA), which may reflect a restricted germination status in these samples. Overall, the higher gibberellin content in the SIt-6, SIt-21, and SIt-17 seeds indicates that gibberellins play an important and complex role in regulating seed germination in these germplasm resources. We conducted seed germination experiments on seeds from different sources to elucidate the relationship between plant hormones and seed germination (Figure 3c,d). The results demonstrated significant differences in germination rates among seeds from different sources. Specifically, seeds from Slt-17 and Slt-1 exhibited the highest germination rates, indicating strong germination potential. Following them were Slt-21, Slt-15, Slt-6, and Slt-19, which also showed high germination rates, albeit slightly lower than those of Slt-17 and Slt-1, but still demonstrating good germination capacity. In contrast, some seeds displayed stronger dormancy, resulting in significantly lower germination rates. Notably, Slt-8, Slt-12, Slt-4, Slt-2, Slt-16, and Slt-13 seeds exhibited strong dormancy, with germination rates far lower than those of the other samples. Of particular interest, Slt-13 seeds exhibited the strongest dormancy, with nearly complete inhibition of germination, which was due to the regulation of abscisic acid in the endogenous hormones of these seeds [17].

## 3. Discussion

*I. indigotica* is an important medicinal plant widely used for heat-clearing, detoxifying, antiviral, and anti-inflammatory treatments. Its roots contain a variety of bioactive compounds, such as alkaloids, anthraquinones, flavonoids, and others, which exhibit significant pharmacological effects [22,23,24]. Research on the metabolites, hormones, and physiological characteristics of *I. indigotica* can enhance its medicinal value, optimize cultivation techniques, and provide a theoretical basis for improving the quality and stability of the medicinal material, thereby promoting its application and development in traditional Chinese medicine. Existing studies have isolated nearly 400 compounds from the roots of *I. indigotica*, including alkaloids [25], organic acids [26], anthraquinones, flavonoids, phenylpropanoids, alcohols, nucleosides, thioglucosides and their metabolites, polysaccharides, trace elements, and more [27]. Research has primarily focused on the root, with studies analyzing the protein composition of *I. indigotica* from a single region [28] and conducting component analyses from the same region [29]. A comparative study on polysaccharides, yields, and alkaloids from 10 varieties of *I. indigotica* grown in two cultivation areas was also conducted [30]. However, no studies have yet been reported on the metabolites, hormones, and their effects on phenotype and germination of *I. indigotica* seeds from different regions in China. Researching the endogenous metabolites and physiological characteristics of *I. indigotica* seeds from different regions is significant for optimizing seed quality, improving planting efficiency, achieving quality breeding, and enhancing the uniformity and adaptability of medicinal plant growth. This study collected 21 germplasm resources of *I. indigotica* from various regions across China, encompassing representative areas with different climate types, including arid, cold, and humid regions [31]. The significant climatic differences among these regions provide a rich ecological background for investigating the advantageous traits of germplasm from different origins. This study found significant differences in seed size, fullness, and morphological uniformity among seeds from different sources. Overall, seeds from northern regions (e.g., Heilongjiang, Daqing) were larger, while seeds from southern regions (e.g., Xingning, Guangdong) were smaller. This trend may be related to environmental adaptation. In northern regions, the cold climate and short growing season pose challenges to early seedling growth due to low temperatures and frost [31]. Larger seeds may have higher nutritional reserves (e.g., higher starch and protein content), which support seedling growth after germination and enhance cold resistance and stress tolerance [32]. In contrast, the warm and humid climate of southern regions [31], along with longer growing seasons, allows seeds to germinate quickly under favorable conditions and potentially undergo multiple reproductive cycles [33]. The differences in seed morphology are not solely caused by environmental factors, the corresponding germplasm genotypes may also play an important role. As a result, smaller seeds may facilitate optimized resource allocation, enabling plants to complete their life cycle more rapidly. Subsequently, we analyzed the storage carbohydrates and organic acid metabolites in seeds from different sources to verify the above conclusions.

In this study, 20 sugar compounds in *I. indigotica* seeds were identified through GC-MS analysis, and significant differences in their accumulation levels were observed among seeds from different sources. Seed samples with high sugar accumulation (SIt-1, SIt-3, SIt-5, SIt-6, SIt-17, and SIt-19) were rich in various sugars, including D-allose, D-(+)-cellobiose, maltitol, α-D-glucopyranoside, D-galactose, and maltose. These sugars not only serve as energy reserves, providing carbon sources for seed germination [34,35], but D-(−)-fructose and galactitol may be involved in osmotic regulation, helping seeds maintain cellular homeostasis under environmental stress [36]. Furthermore, D-ribose and D-(+)-mannose may be associated with signal transduction and cell wall synthesis, enhancing seed physiological activity and environmental adaptability [37,38]. In contrast, seed samples with lower sugar accumulation (e.g., SIt-4, SIt-14, SIt-11) may have insufficient energy reserves due to weaker sugar synthesis or transport capacity, thus affecting seed germination. These results suggest that the sugar metabolism of *Isatis tinctoria* seeds is regulated by both genetic background and environmental factors. In colder or drier environments, plants may synthesize and accumulate more sugars to enhance stress resistance, while in warmer and more humid environments, the sugar accumulation levels in seeds tend to be lower. Additionally, this study identified 10 organic acids, with significant differences in their accumulation levels across different seeds. Seed samples such as SIt-1, SIt-3, SIt-5, SIt-6, SIt-17, and SIt-19 exhibited the highest organic acid content, and were particularly rich in hexadecanoic acid, benzoic acid, propanedioic acid, butanedioic acid, and cinnamic acid. These organic acids play important roles during seed development [39,40]. For example, butanedioic acid, a key intermediate in the tricarboxylic acid (TCA) cycle, promotes seed energy metabolism and improves energy supply, accelerating germination [40]. Benzoic acid and cinnamic acid possess strong antioxidant properties, scavenging free radicals and protecting seed cells from oxidative damage, thereby enhancing environmental adaptability [41]. Moreover, 3-pyridinecarboxylic acid may act as signaling molecules, regulating seed dormancy, germination, and growth development [42]. Based on the accumulation of sugars and organic acids, the seeds from SIt-1, SIt-3, SIt-5, SIt-6, SIt-17, and SIt-19 may have advantages in energy supply, antioxidant capacity, and environmental adaptability, making them more suitable for various ecological environments or demonstrating stronger germination ability under adverse conditions. Additionally, the plant height, width, and 100-grain weight of SIt-18, SIt-20, and SIt-7 were significantly increased. Studies have shown that the significant accumulation of sugars and organic acids can effectively enhance plant biomass [43,44], which is consistent with our results. Correlation analysis further supports these findings, showing positive correlations between metabolites and seed phenotypic characteristics (Figure S1).

The role of plant hormones in seed size and germination ability is multifaceted, with different types of hormones playing unique roles in regulating seed phenotypic traits and germination potential [45]. Through the analysis of seed phenotypic traits (e.g., seed size) and hormone content, we found that plant hormones play a crucial role at various stages of seed development, particularly in regulating seed growth and germination [46]. In terms of hormones regulating seed size, the levels of cytokinins and growth hormones were significantly correlated with seed size [47]. These hormones influence seed development and final size by regulating cell division, elongation, and growth processes [48]. Specifically, the content of cis-zeatin, dihydrozeatin riboside, lignans, and zeatin riboside was higher in SIt-11 and SIt-15 samples, and these seeds exhibited larger volumes (Figure 1), indicating that these hormones play a key role in promoting cell division, expanding cell volume, and accelerating cell elongation. Cis-zeatin and dihydrozeatin riboside, as cytokinin-type hormones, regulate plant growth and development, particularly during the cell division stage [13]. Studies have shown that cytokinins stimulate plant cell division, which in turn promotes the growth of plant organs [14]. In the seeds of SIt-11 and SIt-15, higher levels of these hormones may promote cell division and development of the seed embryo, resulting in larger seeds. Additionally, lignans and zeatin riboside, as growth hormones, also play crucial roles in these processes. Lignans promote plant growth, delay senescence, and enhance the plant’s adaptability to environmental stress, while zeatin riboside is closely associated with cell division, expansion, and tissue growth [49,50]. In contrast, in other seed varieties, the levels of cytokinins and growth hormones were lower, which may contribute to smaller seed sizes (Figure S2). The lower levels of these hormones may inhibit cell division and expansion, limiting seed growth and development, and resulting in smaller seeds. Furthermore, changes in hormone levels may also be closely related to the seed embryo’s developmental rate and nutrient supply, further influencing seed size.

Among the plant hormones that affect seed germination ability, abscisic acid (ABA) and gibberellin (GA) play pivotal roles [51]. ABA is generally considered the main regulatory factor in seed dormancy, as it inhibits seed germination to maintain dormancy, while gibberellin is one of the key hormones promoting seed germination [51]. Gibberellin promotes cell division, elongation, and affects plant embryo growth, thus effectively initiating the germination process [52]. The balance between these two hormones is crucial for seed germination ability. In this study, we found that the seeds of SIt-6, SIt-21, and SIt-17 had higher levels of gibberellin A1 and cis-zeatin, which were closely associated with their higher germination rates (Figure 3). Gibberellin A1 played a critical role in the early stage of seed germination, helping the seeds break dormancy and germinate successfully [53]. Additionally, K-means clustering analysis further revealed the differences in the contents of gibberellin A4, A7, and A9 across different samples, and correlation analysis indicated that these hormones significantly affect seed germination (Figure S2). Higher concentrations of gibberellin A4 and A7 in certain seeds may help promote embryo development and germination, especially under optimal moisture and temperature conditions. Gibberellin A9, as a less-studied member of the gibberellin family, may play a supplementary role in regulating the later stages of seed germination, particularly during the growth phase of the seedling [54].

## 4. Materials and Methods

### 4.1. Sample Collection and Preparation

**Figure 4:**
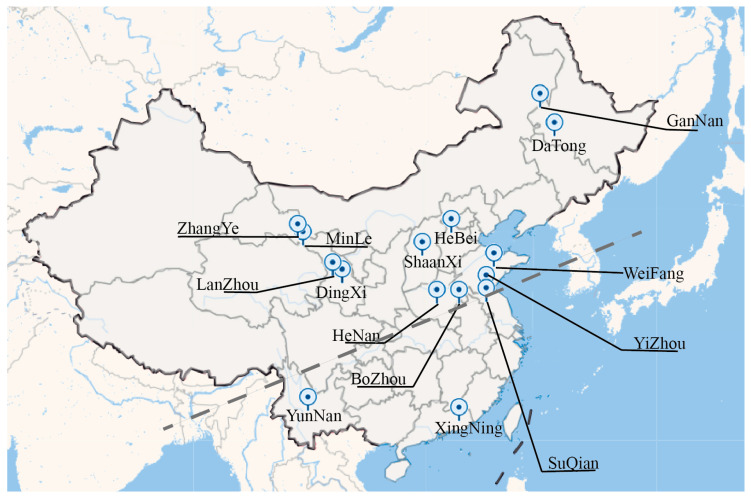
Spatial distribution of the *I. indigotica* seeds samples in China. Blue markers are sampling locations, Grey dotted lines distinguish *I. indigotica* root varieties.

In China, *I. indigotica* is categorized into southern and northern varieties, with different types of *I. indigotica* being harvested from the roots of various plants for medicinal use, primarily sourced from northern germplasm [55]. This study collected 21 germplasm samples of *I. indigotica* from various regions across China to investigate the advantages of different local germplasms. The sampling areas covered multiple representative regions, including Zhangye, Heilongjiang, Lanzhou, Weifang, Shanxi, Hebei, Henan, Bozhou, Yunnan, Xingning, Suqian, Yizhou, and Minle (Figure 4). The climatic conditions of these regions vary significantly. Zhangye, located in the northwest of Gansu Province, is characterized by an arid climate, suitable for drought-resistant plants. Heilongjiang has a cold and humid climate, where the germplasms exhibit strong cold resistance and well-developed root systems. Lanzhou, situated in central Gansu Province, experiences a temperate semi-arid climate. Weifang, in eastern Shandong Province, has a mild and humid climate. Shanxi is predominantly semi-arid, while Hebei and Henan have mild and humid climates. Bozhou, in central Anhui Province, enjoys a warm and humid climate with fertile soils, conducive to the growth of various plants. Yunnan, in southwestern China, features a warm and humid climate with abundant rainfall, ideal for the growth of various crops. Xingning, located in central Guangdong Province, has a warm and humid climate. Suqian, in northern Jiangsu Province, also has a warm, humid climate. Yizhou and Minle have warm and humid climates with abundant rainfall. The study of these germplasms will provide valuable insights into the adaptive traits and potential advantages of the I. indigotica resources from these diverse climatic regions, offering scientific guidance for germplasm utilization and breeding. The collected samples were verified against the official database of the Flora of China (https://www.iplant.cn/frps 03 February 2025) and assigned corresponding catalog numbers to confirm their species classification and origin (Table 1). This verification provides a reliable foundation for subsequent analyses. The authentication of the specimens significantly enhances the credibility of the study. 

The samples we collected were primarily from the northern regions. However, to ensure a diverse range of samples and to enhance the completeness of the study, we also included four southern varieties of *I. indigotica* from locations such as Suqian in Jiangsu Province, Xingning in Guangdong Province, and Yunnan Province. This allows for comparisons in the subsequent experiments. The sampling period was from June to July 2023. After retrieving the seeds, they were first rinsed under running water to remove impurities. Then, they were placed in an environment at 25 °C for 2 days to dry before conducting subsequent experiments.

### 4.2. Measurement of Seed Phenotypic Parameters

After collecting seeds from the same plant at the same maturity stage in different regions and drying them at 25 °C for 2 days, we randomly selected 10 seeds within each group of seeds from the same source to measure phenotypic parameters. A total of 10 seeds from each group were placed on a checkered paper by turn for phenotypic photography at the same position. Their length and width were measured and recorded using vernier calipers (accuracy 0.01 mm). Additionally, 100 seeds were as same selected and weighed. All of the above process was repeated 3 times to ensure that the data was as accurate as possible.

### 4.3. Sample Extraction and Metabolite Profiling of I. indigotica Seed from 21 Origins in China

A total of 90 mg of dried sample was homogenized with a steel ball at 60 Hz for 2 min. Cold methanol (560 μL) and internal standard (2-Chloro-L-phenylalanine,0.3 mg/mL) were added, followed by sonication and the addition of chloroform (300 μL). The supernatant was collected, dried, and reconstituted with methoxylamine hydrochloride (400 μL). After incubation, N, O-bis (trimethylsilyl) trifluoroacetamide and trimethylchlorosilane were added for GC-MS analysis after derivatization. The GC-MS data were obtained using an Agilent 7890-5977B instrument (Agilent, Santa Clara, CA, USA). In the detection method, the programmed warming parameters were set based on Li et al. [55]. Mass spectra were recorded while scanning from 50–600 *m*/*z*. The above process was repeated 3 times to ensure that the data was as accurate as possible, and important metabolites were screened using *t*-test combinatory approaches (*p* < 0.05).

### 4.4. Analysis of Endogenous Hormones in I. indigotica Seed from 21 Origins in China

A total of 120 mg (dried weight) of the sample was homogenized under cryogenic conditions (liquid nitrogen milling at 50 Hz, 30 s, two cycles). Approximately 100 mg of the cryogenically processed sample was precisely weighed and combined with 1000 μL of extraction solution (methanol-acetonitrile 1:1 *v*/*v* containing isotope-labeled internal standards at 2 ng/mL, pre-chilled to −40 °C). Following 2-h incubation at −40 °C, the mixture was subjected to centrifugation at 12,000 rpm (4 °C, 15 min). Subsequently, 900 μL of supernatant was collected and lyophilized in a temperature-controlled spin dryer (4 °C). The dried residue was reconstituted with 90 μL of 50% aqueous methanol, followed by sequential vortex mixing (1 min), ultrasonication (120 s), and additional vortex mixing (1 min) to ensure complete dissolution. The reconstituted sample was then processed as follows: 90 μL of 50% aqueous methanol (*v*/*v*) was added, followed by sequential vortex mixing (1 min), ultrasonication (120 s), and additional vortex mixing (1 min). The mixture was centrifuged at 12,000 rpm (4 °C, 10 min), after which 80 μL of supernatant was transferred to a new polypropylene tube and recentrifuged under identical conditions. Finally, 70 μL of the clarified supernatant was aliquoted into a pre-labeled chromatographic vial for LC-MS analysis. Separation was performed on an ExionLC™ AD UHPLC System (SCIEX) equipped with a Kinetex C18 column (2.1 × 100 mm, 2.6 μm; Phenomenex, Torrance, CA, USA). The mobile phase consisted of the following: Phase A: 0.1% (*v*/*v*) formic acid in water; Phase B: 0.1% (*v*/*v*) formic acid in methanol. The gradient elution program was 2%B at 0–1 min, 2–95% B at 1–6 min, 95% B at 6–8 min, 95–2% B at 8–8.1 min, and 2% B at 8.1–10.1 min. The column compartment was maintained at 25 °C with a flow rate of 0.25 mL/min. A 2 μL injection volume was employed for all analyses. The above process was repeated 3 times to ensure that the data was as accurate as possible.

The MRM (Multiple Reaction Monitoring) parameters for each of the targeted analytes were optimized using flow injection analysis by injecting the standard solutions of the individual analytes into the API source of the mass spectrometer. Several most sensitive transitions were used in the MRM scan mode to optimize the collision energy for each Q1/Q3 pair (Table S1). Among the optimized MRM transitions per analyte, the Q1/Q3 pairs that showed the highest sensitivity and selectivity were selected as ‘quantifier’ for quantitative monitoring. The additional transitions acted as ‘qualifier’ for the purpose of verifying the identity of the target analytes. Analyst (1.7.3, SCIEX) (SCIEX, Framingham, MA, USA) was employed for MRM data acquisition.

### 4.5. Seed Germination Experiment

The selected seeds were soaked in warm water at 30 °C for 12 h to break dormancy. A total of 30 seeds were randomly placed into petri dishes, and 15 mL of deionized water was added to each dish. The dishes were then placed in an incubator at a temperature of 25 °C with a light/dark cycle of 14/10 h. The light intensity was set at 4000 lx, and the seeds were rehydrated with deionized water every 2 days. The number of germinated seeds was recorded daily, and the germination test was concluded after 5 days. All seeds with radicle protrusion were considered germinated.

### 4.6. Statistical Analysis

The graphs were plotted using Origin Pro 2024 (OriginLab, Northampton, MA, USA) and R software (version 4.3.3). Correlation analysis, significance analysis, and K-means clustering were performed using the SPSS system (SPSS 27.0, SPSS Inc., Chicago, IL, USA). Data were subsequently subjected to one-way analysis of variance (ANOVA) and Student’s *t*-test (*p* < 0.05).

## 5. Conclusions

In this study, we validated the feasibility of using data fusion to examine the differences in *I. indigotica* seeds from various regions through GC-MS and LC-MS, focusing on phenotypes, physical parameters, compounds, and hormones. The results indicated that the quality of Chinese bluebell roots from the north was generally superior to that from the south, and that small leaves were more advantageous than large leaves within the same regions. Data analysis revealed that the accumulation of compounds detected by GC-MS was generally consistent with the levels of hormone accumulation, which also correlated with germination rates, seed phenotypes, and physical parameters. However, further studies on the effects of environmental factors, genotypes interaction and their interactions on the quality of *I. indigotica* are needed in the future to enhance cultivation practices and conserve wild populations. Similarly, our study provides a scientific basis for further ensuring and identifying high-quality *I. indigotica* germplasm.

## Figures and Tables

**Table 1:** Seed numbers and geographic affiliation. Collection name stands for the nickname used at the time of collection, Serial number stands for the code number sorted after collection, GPS stands for Global Positioning Unit, Category stands for whether the collection occurred in northern Banlangen (dried root of *I. indigotica*), and voucher number indicates the voucher number of the *I. indigotica* seed in Chinese Virtual Herbarium.

No.	Collection Name	Serial Number	Province	GPS Coordinates	Category	Voucher Number
1	SuQian	SIt-1	JiangSu	N 118.28° E 33.96°	*Isatidis Radix*	NAS NAS00113843
2	HeBei B.leaves	SIt-2	HeBei	N 116.57° E 39.92°	*Isatidis Radix*	IBSC 0137007
3	HeNan	SIt-3	HeNan	N 113.75° E 34.77°	*Isatidis Radix*	PE 01055155
4	BoZhou	SIt-4	AnHui	N 115.78° E 33.85°	*Isatidis Radix*	WUK 0004816
5	MinLe	SIt-5	GanSu	N 100.81° E 38.43°	*Isatidis Radix*	WUK 0069675
6	HeBei S.leaves	SIt-6	HeBei	N 114.53° E 38.04°	*Isatidis Radix*	PE 01055154
7	YunNan B.Leaves	SIt-7	YunNan	N 102.71° E 25.05°	*Isatidis Radix*	KUN 0498901
8	YunNan S.leaves	SIt-8	YunNan	N 102.71° E 25.05°	*Isatidis Radix*	KUN 0498903
9	GanNan	SIt-9	GanSu	N 123.51° E 47.92°	*Isatidis Radix*	KUN 0498912
10	LanZhou	SIt-10	GanSu	N 103.83° E 36.06°	*Isatidis Radix*	WUK 0081682
11	DingXi S.leaves	SIt-11	GanSu	N 104.59° E 35.61°	*Isatidis Radix*	QYTC QYTC0003047
12	DingXi B.leaves	SIt-12	GanSu	N 104.59° E 35.61°	*Isatidis Radix*	QYTC QYTC0005994
13	WeiFang	SIt-13	ShanDong	N 119.16° E 36.71°	*Isatidis Radix*	GXMG GXMG0009684
14	DaTong B.leaves	SIt-14	ShanXi	N 124.92° E 46.11°	*Isatidis Radix*	SXTCM SXTCM0004019
15	XingNing	SIt-15	GuangDong	N 115.73° E 24.14°	*Isatidis Radix*	SZG SZG00028974
16	ZhangYe B.leaves	SIt-16	GanSu	N 100.45° E 38.92°	*Isatidis Radix*	PE 01055158
17	ShanXi S.leaves	SIt-17	ShanXi	N 112.58° E 37.81°	*Isatidis Radix*	WUK 0321714
18	ZhangYe S.leaves	SIt-18	GanSu	N 100.45° E 38.92°	*Isatidis Radix*	QYTC QYTC0003048
19	DaTong S.leaves	SIt-19	ShanXi	N 124.92° E 46.11°	*Isatidis Radix*	WUK 0324249
20	YiZhou B.leaves	SIt-20	ShanDong	N 118.36° E 35.10°	*Isatidis Radix*	NAS NAS00113877
21	YiZhou S.leaves	SIt-21	ShanDong	N 118.36° E 35.10°	*Isatidis Radix*	NAS NAS00113884

## Data Availability

Data are contained within the article and Supplementary Materials.

## Supplementary Materials

The following supporting information can be downloaded at: https://www.mdpi.com/article/10.3390/plants14071096/s1, Figure S1: Correlation analysis of 15 different regions of *I. indigotica* seeds’ length, width, weight, and seed germination with sugars and organic acids. “*” indicates *p* < 0.05, statistically significant, “**” indicates *p* < 0.01 and “***” indicates *p* < 0.001, statistically significant. Figure S2: Correlation analysis of 15 different regions of *I. indigotica* seeds’ length, width, weight, and seed germination with hormones. “*” indicates *p* < 0.05, statistically significant, “**” indicates *p* < 0.01 and “***” indicates *p* < 0.001, statistically significant. Table S1: Mass spectrometry detection parameters for each hormone in the MRM scan mode.
