# Supplementary material for: Phenotypic Traits, Hormonal Distribution, and Metabolite Profiling of Isatis indigotica Seeds from 21 Samples in China: A Traditional Chinese Medicinal Herb
Source: Plants (Basel). 2025 Apr 1;14(7):1096. doi: 10.3390/plants14071096 (PMC11991228; doi:10.3390/plants14071096)
Supplement: Supplementary file 1 [file plants-14-01096-s001.zip › Supplementary Material.pdf]

Figure S1: Correlation analysis of 15 different regions of indigowoad root seeds' length, width, weight and germination with sugars and organic acids. "*" indicates $p < 0.05$ statistically significant, "**" indicates $p < 0.01$ and "***" indicates $p < 0.001$, statistically significant.

Figure S2: Correlation analysis of 15 different regions of indigowoad root seeds' length, width, weight and germination with hormones. "*" indicates $p < 0.05$ statistically significant, "**" indicates $p < 0.01$ and "***" indicates $p < 0.001$, statistically significant.

Figure S1

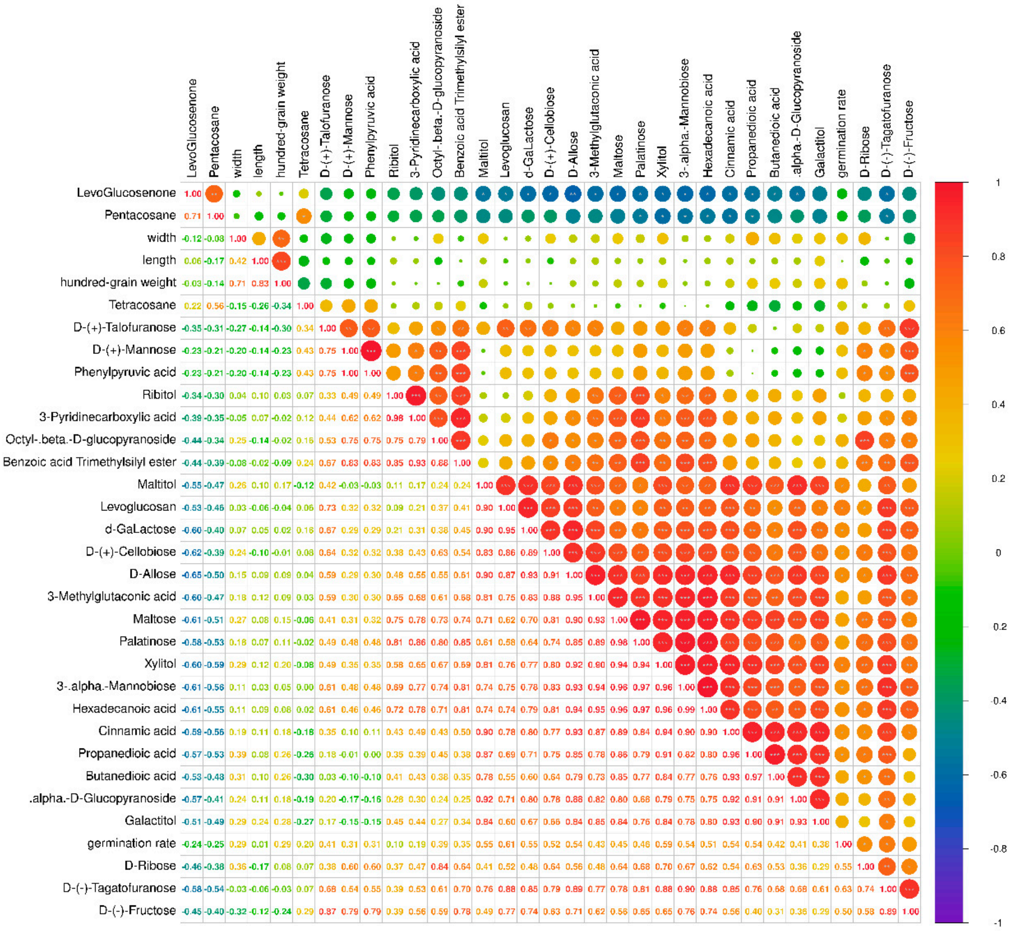
LevoGlucosenone
Pentacosane
width
length
hundred-grain weight
Tetracosane
D-(+)-Talofuranose
D-(+)-Mannose
Phenylpyruvic acid
Ribitol
3-Pyridinecarboxylic acid
Octyl-.beta.-D-glucopyranoside
Benzoic acid Trimethylsilyl ester
Maltitol
Levoglucosan
d-GaLactose
D-(+)-Cellobiose
D-Allose
3-Methylglutaconic acid
Maltose
Palatinose
Xylitol
3-.alpha.-Mannobiose
Hexadecanoic acid
Cinnamic acid
Propanedioic acid
Butanedioic acid
.alpha.-D-Glucopyranoside
Galactitol
germination rate
D-Ribose
D-(-)-Tagatofuranose
D-(-)-Fructose
1
0.8
0.6
0.4
0.2
0
-0.2
-0.4
-0.6
-0.8
-1

Figure S2

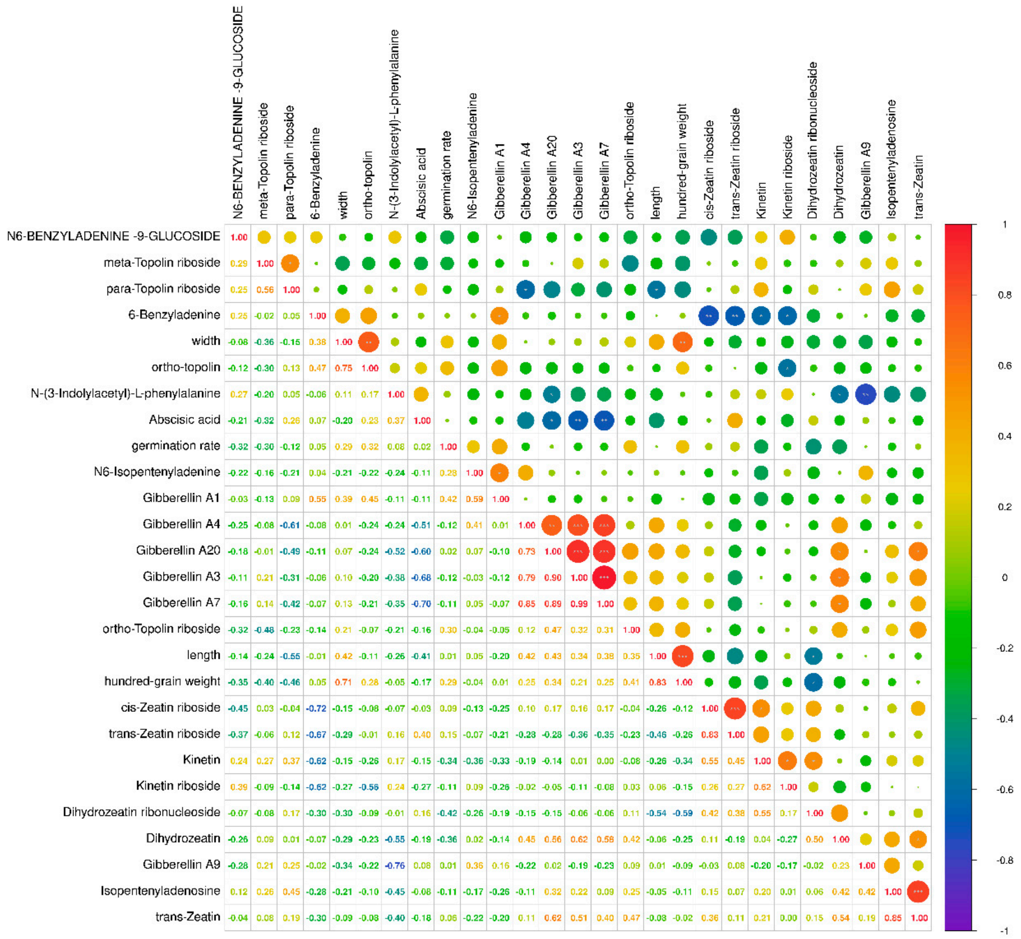

N6-BENZYLADENINE -9-GLUCOSIDE
meta-Topolin riboside
para-Topolin riboside
6-Benzyladenine
width
ortho-topolin
N-(3-Indolylacetyl)-L-phenylalanine
Abscisic acid
germination rate
N6-Isopentenyladenine
Gibberellin A1
Gibberellin A4
Gibberellin A20
Gibberellin A3
Gibberellin A7
ortho-Topolin riboside
length
hundred-grain weight
cis-Zeatin riboside
trans-Zeatin riboside
Kinetin
Kinetin riboside
Dihydrozeatin ribonucleoside
Dihydrozeatin
Gibberellin A9
Isopentenyladenosine
trans-Zeatin
1
0.8
0.6
0.4
0.2
0
-0.2
-0.4
-0.6
-0.8
-1
